# Paratuberculosis: A Potential Zoonosis and a Neglected Disease in Africa

**DOI:** 10.3390/microorganisms8071007

**Published:** 2020-07-05

**Authors:** Julius Boniface Okuni, Sören Hansen, Kamal H. Eltom, ElSagad Eltayeb, Ahmad Amanzada, Joseph Amesa Omega, Claus Peter Czerny, Ahmed Abd El Wahed, Lonzy Ojok

**Affiliations:** 1 College of Veterinary Medicine, Animal Resources and Biosecurity (COVAB), Makerere University, P. O. Box 7062 Kampala, Uganda; jbokuni@gmail.com (J.B.O.); lonzyo@yahoo.com (L.O.); 2 Division of Microbiology and Animal Hygiene, University of Goettingen, D-37077 Goettingen, Germany; hansensoer@gmail.com (S.H.); cczerny@gwdg.de (C.P.C.); 3 Unit of Animal Health and Safety of Animal Products, Institute for Studies and Promotion of Animal Exports, University of Khartoum, 13314 Shambat, Khartoum North, Sudan; keltom@daad-alumni.de; 4 Faculty of Medicine, Alneelain University/Ibn Sina Specialised Hospital Khartoum, 11112 Khartoum, Sudan; sagadgady@yahoo.com; 5 Department of Gastroenterology and Gastrointestinal Oncology, University Medical Centre Goettingen, D-37075 Goettingen, Germany; ahmad.amanzada@med.uni-goettingen.de; 6 School of Agriculture and Biotechnology, Department of Animal Science and Management, University of Eldoret, P. O. Box 1125-30100 Eldoret, Kenya; josephomega8@gmail.com; 7 Institute of Animal Hygiene and Veterinary Public Health, University of Leipzig, D-04103 Leipzig, Germany

**Keywords:** *Mycobacterium avium* subspecies *paratuberculosis*, paratuberculosis, idiopathic inflammatory bowel disease, Africa

## Abstract

The *Mycobacterium avium* subspecies *paratuberculosis* (MAP) is the causative agent of paratuberculosis, which is an economically important disease of ruminants. The zoonotic role of MAP in Crohn’s disease and, to a lesser extent, in ulcerative colitis, the two major forms of idiopathic inflammatory bowel disease (IIBD), has been debated for decades and evidence continues to mount in support of that hypothesis. The aim of this paper is to present a review of the current information on paratuberculosis in animals and the two major forms of IIBD in Africa. The occurrence, epidemiology, economic significance and “control of MAP and its involvement IIBD in Africa” are discussed. Although the occurrence of MAP is worldwide and has been documented in several African countries, the epidemiology and socioeconomic impacts remain undetermined and limited research information is available from the continent. At present, there are still significant knowledge gaps in all these areas as far as Africa is concerned. Due to the limited research on paratuberculosis in Africa, in spite of growing global concerns, it may rightfully be considered a neglected tropical disease with a potentially zoonotic role.

## 1. Introduction

Paratuberculosis, also called Johne’s disease (JD), occurs in a wide range of domestic and wild ruminants. It is caused by *Mycobacterium avium* subspecies *paratuberculosis* (MAP). The disease manifests as a granulomatous inflammation involving the intestinal mucosa and the mesenteric lymph nodes before involving other lymph nodes [1]. It is a classic example of a protein-losing enteropathy, where the inflammation of the intestinal mucosa renders the absorptive epithelium incapable of adequate nutrient absorption while allowing the escape of fluid and nutrients out through the feces [2]. This state of affairs leads to a malabsorptive and secretory diarrhea, which manifests clinically as a projectile diarrhea in cattle or loosely formed feces, as seen in sheep and goats. The results of this are reduced weight gain, reduced milk, meat or wool production, emaciation and submandibular edema in the terminal stages [3].

Concerns about the disease stem from its devastating impact on dairy cattle and other farmed ruminants, in addition to the public health concern due to the possible role of MAP in the related enteric disorders of man, such as idiopathic inflammatory bowel disease (IIBD), Crohn’s disease (CD) and ulcerative colitis [4].

Paratuberculosis has been described in the United States of America as ‘a hidden threat’ [5], a ‘silent slayer’ [6] and the ‘most important single etiology of disease of cattle in the coming decades’ [7]. Its prevalence among the dairy herds in the United States of America has been reported to have increased within just 10 years, from 21.6% in 1996 to 91.1% in 2007 [8]. In spite of all these, research funding on paratuberculosis has been limited, even in developed countries [4,9]. There are few countries with a mandatory paratuberculosis control program, even fewer with an eradication program, while the majority of countries do not even have research programs on this disease [10].

**Figure 1:**
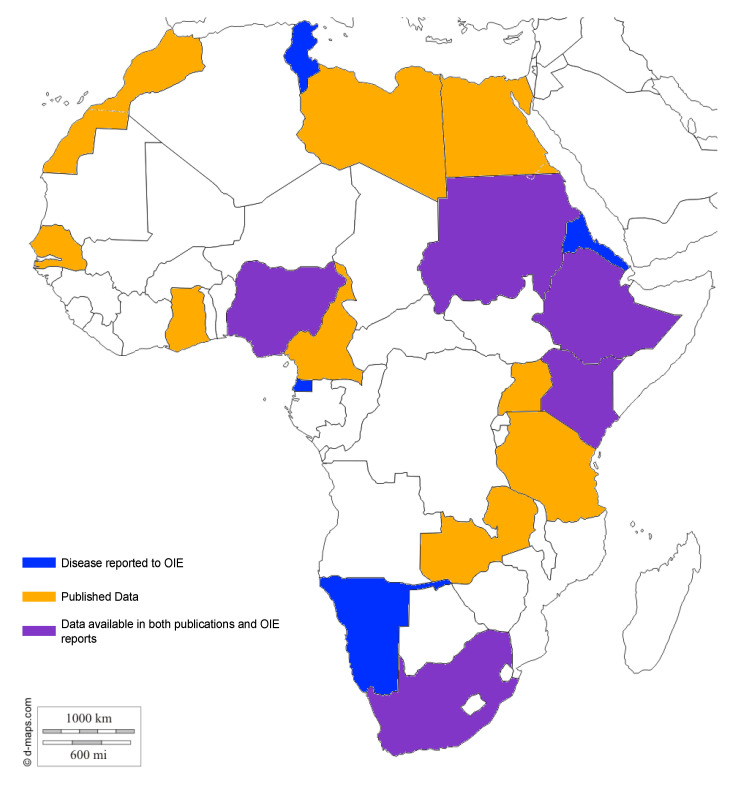
Map of the prevalence of paratuberculosis in Africa as reported to the World Organization for Animal Health, formerly the Office International des Epizooties (OIE) and/or in publications.

Although the occurrence of paratuberculosis has been documented in many African countries, they are among those countries from which limited information regarding the prevalence, control or research is available (see Figure 1 and Table 1). This is attributable to the general lack of awareness and limited research on animal diseases in these countries and uncoordinated reporting of cases of the disease [11,12]. In most developing countries, there is little awareness about the occurrence of this disease in the livestock population. Even where the disease is well documented, it is often at the bottom of the list of priorities for research and control [11] and is therefore neglected. The World Health Organization (WHO) and the Food and Agriculture Organization of the United Nations (FAO) define neglected tropical diseases as ‘a diverse group of communicable diseases that prevail in tropical and sub-tropical conditions in 149 countries, affecting more than one billion people and costing developing economies billions of dollars every year [13]. The case for paratuberculosis will, therefore, require a demonstration that it affects billions of people and cost billions of dollars. Such a demonstration will require a lot of research on and publicity about the disease. Unfortunately, the insidious nature of the disease, the difficulty of early diagnosis and the presence of so many other tropical and sub-tropical diseases that can be confused with paratuberculosis, coupled with the current dearth of research into this disease in Africa, make proving paratuberculosis as a serious threat a daunting challenge. The disease will thus continue to be neglected and continue to spread. In short, from a research perspective, paratuberculosis is a neglected disease in Africa.

## 2. Pathogen Biology

The causative agent of paratuberculosis, MAP, is an acid-fast bacterium belonging to the *Mycobacterium avium* intracellular complex (MAIC). It is dependent on externally provided sources of mycobactin, an iron chelating molecule, for its growth; a characteristic that distinguishes most of its strains from other mycobacteria [44]. Within the animal host, the bacteria multiplies in macrophages following the disabling of phagocytosis and intracellular killing by the cells [1]. This leads to the formation of granulomas as the body tries further to destroy the pathogen. MAP prefers an intracellular environment that is rich in iron [45] calcium and pyruvate [46]. MAP is believed to subsist in the environment in microfilms like other mycobacteria [47]. Free-living amoebas and some invertebrate hosts may also carry the organism [48] within the environment. There is evidence that MAP also has a dormancy response, which enables it to survive in the environment for long periods of up to two years or more [49]. MAP is surrounded by a complex tripartite lipid-rich cell wall that enables it to persist in the environment and contributes to its resistance to low pH, high temperatures and chemical agents [50]. Hence, once an environment is contaminated, it will take a very long period before animals can be safely introduced into that environment. These factors create great challenges for the eradication of paratuberculosis in any country. Environmental factors such as humid acid soils have been proved to foster the persistence of MAP in the environment [51]. It is thus recommended that the addition of lime to the soil should be attempted to reduce the persistence of MAP in the farm environment.

In the laboratory, MAP is a fastidious organism that requires special media in which it grows very slowly, taking a minimum of 8 weeks for colonies to appear or up to one year upon primary isolation. Growth will be realized on Herrold’s egg yolk medium, modified Lowenstein–Jensen media, Middlebrook agar 7H9 and 7H10, all supplemented with Mycobactin J and pyruvate. Although dependence on mycobactin has been used as a cardinal criterion for the confirmation of acid-fast bacilli such as MAP, there are reports indicating that some strains of MAP do not need mycobactin [52], but this needs to be researched further. It has also been reported that some strains will not grow when pyruvate is added to the media, while other strains require pyruvate. These factors make the isolation of MAP very complicated [46]. MAP isolates from CD are even more difficult to grow and current opinions suggest that they exist in cell wall-deficient forms, spheroplasts or L-forms [53].

## 3. Host Diversity

Although MAP has traditionally been known to infect cattle, goats and sheep, the organism has been reported to cause disease in camels, horses and pigs, among domestic animals, and in deer, springbok, elk, bison, rabbits, dogs, foxes, stoat, badgers, nonhuman primates, ravens and other wild animal species. It is possible that the list of susceptible hosts of MAP will continue to grow [7]. A granulomatous cellulitis has been reported in man following accidental inoculation [54]. A case of MAP infection in an acquired immune deficiency syndrome (AIDS) patient has been reported [55]. Most of these reports are from studies in Europe, the Americas and Asia. In Africa, Miller et al. [29] recently reported a case of paratuberculosis causing disease in dogs in South Africa, while Otchere et al. [19] reported that MAP comprised some of the Nontuberculous Mycobacteria (NTMs) isolated from human patients with pulmonary disease. The high burdens of Human Immunodeficiency Virus (HIV)/AIDS, several other immunocompromising diseases and malnutrition in many parts of Africa put large sections of the population at risk of contracting opportunistic infections such as MAP. Health facilities in Africa do not test for MAP in humans and CD is largely unknown, even among health professionals. This is despite the fact that the incidence of chronic, intractable diarrhea that is refractive to treatment is high in the population [12]. 

## 4. Ecology of MAP Infection

Infected animals’ feces contaminate pastures, soil and water [7], followed by a prolonged persistence of the organism in the environment because of its tolerance to harsh environmental conditions [51,56]. Whittington et al. [49] found that MAP survives up to 35 weeks in dry, fully shaded environments and for a shorter period (32 weeks) in un-shaded locations. It survives longer in acidic than in alkaline soils [57] and in soils rich in calcium and iron [58,59]. Heavy rains and flooding contribute to the spread of the pathogen across vast areas. Moreover, animals from different households grazing on communal grounds and animals transported on foot for long distances contribute to the spread of MAP [12]. These factors, which may affect the spatial distribution of the disease, are yet to be investigated in Africa. *M. avium* is known to be fairly resistant to chlorine water treatment and has been recovered from treated water [60,61]. MAP is able to survive for extended periods under simulated water treatment conditions. MAP has been recovered from estuaries of rivers that drain the areas around affected farms [62]. Survival on the surfaces of watering troughs through biofilm formation and environments around farms has also been documented [63,64]. These facts make it difficult to control the spread of the disease and raise questions of the possibility of food and water contamination along the human food chain. The presence of MAP in environmental samples, water estuaries around affected farms and the possible infection of humans and other animals will need to be investigated. There is still confusion and controversy regarding whether MAP survives pasteurization under standard commercial pasteurization or not [65]. Several reports have shown that MAP survives commercial pasteurization [66,67,68], most recently in calf milk replacer [69], while other studies suggest that MAP is killed by pasteurization [5]. The critical factors appear to be time and holding temperatures, which differ depending on the commercial pasteurization process. The possibility that commercial pasteurization does not destroy MAP is of public health concern in the event that MAP is proven to be a cause of IIBD.

## 5. Paratuberculosis in Livestock and Wildlife

Although wildlife is very important in the economy of Sub-Saharan Africa, investigations of MAP in African wildlife have not been undertaken yet. Fechner et al. [28] diagnosed MAP in hyraxes imported from South Africa to Germany, but no systematic investigation has been carried out on MAP infection in wildlife in Africa. Anecdotal records in Uganda indicate that lesions characteristic of MAP have been documented in an impala (Ojok, Personal communication). A lot needs to be done to establish the state of MAP infection in wildlife in all countries in Africa. Due to human encroachment with their domestic animals into game reserves and parks, the possibility of cross-infection between wild and domestic ruminants is real and needs to be investigated.

## 6. The Role of MAP in Idiopathic Inflammatory Bowel Disease and Related Diseases 

Evidence of the causal relationship between MAP and CD has for long been debated. This arose because of the similarity between clinical signs and pathological lesions of CD and Johne’s disease [70]. In one such analysis, it was stated that MAP fulfils four of the six criteria for causation of a disease with respect to CD [71]. Several studies have associated MAP with IIBD, which comprises CD and ulcerative colitis (UC) [70,72,73]. The isolation of MAP from the ileal tissues of patients with CD, its identification in the intestinal tissues of some CD patients, as well as its culture from peripheral blood monocytes and from breast milk of CD patients [71,74,75,76,77,78] have provided a strong association between MAP and CD. The results of a blind study from three independent laboratories, which confirms a higher prevalence of viable MAP in CD patients than in controls [79], the concurrent increase in the herd prevalence of JD and CD over the same period in the USA [8,80] and the reported remission and cure of CD patients treated with antibiotics against MAP [81,82] lend more credence to the theory that MAP could be a cause of CD. In one study [83], an association between the presence of MAP DNA in the intestinal biopsy tissues of CD patients and the consumption of unpasteurized dairy products in Brazil was found. In view of this possible connection, the finding of MAP in pasteurized dairy products is believed to raise a serious public health concern [68]. Furthermore, a link has also emerged showing that similar genetic polymorphisms are found in CD patients and MAP [84,85,86]. MAP would become one of the most important public health concerns of the future if it is confirmed that it is the cause of IIBD (CD, UC).

## 7. Current Status of Idiopathic Inflammatory Bowel Disease and Related Diseases in Africa

IIBD and CD were long considered to be very rare in Africa, especially in the black African population [87]. However, recent studies indicate that with the improvement of diagnoses, such as the use of colonoscopy in hospitals, like in Egypt and Sudan, IIBD is no longer as rare as was initially thought [88,89]. A study in Egypt showed that UC was responsible for 22% of the indications for colonoscopy, while CD is responsible for 3% of such indications alongside colorectal cancer (15%), hemorrhoids (18%) among others [89]. A retrospective study in Sudan over a 10-year period found that there were six times more UC cases compared with CD and concluded that IIBD is not rare in the country [90]. In other African countries, cases of CD have been reported, e.g., in South Africa [91,92], Kenya [87], Senegal [93] and the Cote D’Ivoire [94]. The low incidences of IIBD cases have been attributed to a limited diagnostic capacity and difficulties in distinguishing the conditions from tuberculosis and schistosomiasis of the bowel, which are quite common [87]. In our view, another reason for the reported low incidence of IIBD in Africa is due to a lack of population-wide studies of these supposedly rare conditions. A recent study in Egypt has shown that some of the humans tested were positive for MAP antibodies, but concluded that the correlation between MAP seropositivity and CD was weak [90]. It was not known how the human population acquired this seropositivity in the above-cited study. Similar findings have been documented in India [72,95]. More studies need to be done to investigate this phenomenon in Egypt as well as in other African countries. There is, however, a corresponding overlap between antibodies of MAP in humans and the prevalence of MAP infection in livestock in India [72]. It is equally necessary that precautionary measures are taken to limit human exposure to MAP wherever this pathogen occurs in Africa and elsewhere in the world [96]. It is of great importance that, as research into the prevalence, virulence and pathogenesis of MAP in Africa is encouraged, emphasis should also be directed towards investigations into the possible MAP involvement in CD and UC in Africa.

## 8. Socio-Economic Impact of MAP in Africa

Because of the limited studies thus far undertaken in Africa, there is no estimate of the socioeconomic impact of paratuberculosis in Africa at the moment, either on livestock or on man. A report from Kenya stated that, whereas 25% of the income of small scale dairy farmers in Kenya came from milk sales, a fifth of their total income was spent on animal inputs, such as drugs, veterinary services and feed supplements [97]. Poor knowledge about paratuberculosis may have led the farmers to inadvertently exacerbate its spread through practices like calves suckling, communal grazing and resistance to cull sick animals due to traditional restrictions [21]. Following the iceberg concept, the detection of one positive or clinically sick animal suggests that there are several times more sub-clinically affected animals that will, with time, show clinical symptoms. Economic losses include the costs of veterinary care of animals with frequent diarrheal episodes, poor growth rates and weight gain, culling, etc., as has been documented in several studies in developed countries [12].

## 9. Control and Management

As with economic impacts, there are no reports on the control of paratuberculosis in Africa. This is not just for paratuberculosis alone, but for many other diseases as well. The control of paratuberculosis remains therefore the responsibility of the farm owner with the help of veterinarians and other animal health workers. Ironically, JD is a notifiable disease not just according to the World Organization for Animal Health, formerly the Office International des Epizooties (OIE) [98], but also in many African countries. However, there is very little governmental effort to detect and control it. Knowledge about the disease is almost non-existent [97] and both farmers and veterinarians only get to see the devastation of the disease in the terminal stages or at post-mortem after trying, in vain, to treat the animals with anthelmintics and antibiotics. It is, therefore, of great importance that sufficient baseline information be generated about the disease and provided to both governments and farmers to make sound policies and decisions, respectively, on paratuberculosis control and management.

## 10. Future Research on MAP in Africa

Future studies need to focus on the epidemiology of the disease, distribution, risk factors and the effects of environmental and host factors that affect the occurrence and possible resistance or tolerance among species and breeds. Other areas of study should include the molecular epidemiology of different strains and the possibility of infection across host species and possible differences in virulence, to identify strains that can be used for vaccination. The need to find new and better diagnostics is a task that the global research community is still grappling with and African scientists can also make contributions in this area. The possible link between MAP and IIBD/CD as well as the seropositivity of the human population to MAP antigens require clarification. Finally, and most importantly, the economic impact of paratuberculosis needs to be determined alongside cost–benefit analyses for instituting control programs.

## 11. Conclusions

MAP poses a great challenge to the global livestock industry and is currently insidiously spreading in Africa. Moreover, it could have possible impacts on human health across the continent. Given the fact that any known policies on this pathogen and most of the vital information required for instituting control and policy formulation are deficient, Africa remains a shadowy continent as far as this pathogen is concerned; therefore, paratuberculosis is, without any doubt, a neglected disease with possible zoonotic involvement in the African context. Given that the few studies undertaken on this disease have shown unfailing occurrence in several African countries, the disease is marching ahead of all stakeholders in the animal industry and needs to be closely studied. More attention in terms of funding and research needs to be given to Johne’s Disease in African countries.

## Figures and Tables

**Table 1:** Reports on *Mycobacterium avium* subspecies *paratuberculosis* (MAP) in African counties.

Country	Species	No. of Reported Cases	Method			Reference	Reported to OIE
			PCR	Culture	Ab Detection		
Algeria	n.d.						
Angola	n.d.						
Benin	n.d.						
Botswana	n.d.						
Burkina Faso	n.d.						
Burundi	n.d.						
Cabo Verde	n.d.						
Cameroon	Cattle	132		x	x	[14]	n.d
Central African Republic	n.d.						r.n.
Chad	n.d.						r.n.
Comoros	n.d.						
Democratic Republic of the Congo	n.d.						
Republic of the Congo	n.d.						
Cote d’Ivoire	n.d.						
Djibouti	n.d.						r.n.
Egypt	Cattle	17	x			[15]	r.n.
		75		x		[16]	
Equatorial Guinea	n.d.						s (2013)
Eritrea	n.d.						+ (2008)
Ethiopia	Cattle	5		x		[17]	+ (1996)
					x	[18]	
Gabon	n.d.						
Gambia	n.d.						
Ghana	human	13	x			[19]	r.n.
Guinea	n.d.						
Guinea Bissau	n.d.						
Lesotho	n.d.						
Liberia	n.d.						
Libya	n.d.						
Kenya	Cattle		x	x	x	[20,21]	+ (2015)
	Camel/Cattle	102/69			x	[22]	
	Sheep		x	x	x	[21]	n.d.
Madagascar	n.d.						r.n.
Malawi	n.d.						
Mali	n.d.						
Mauritania	n.d.						
Mauritius	rusa deer	351			x	[23]	+ (1995)
		2	x				
Morocco	sheep	180	x	x		[24]	n.d.
		56		x	x	[25]	
		2		x		[26]	
Mozambique	n.d.						
Namibia	n.d.						+ (1988/2004)
Niger	n.d.						
Nigeria	Cattle	n.d.				[11]	+ (2017)
Rwanda	n.d.						
Sao Tome and Principe	n.d.						
Senegal	Cattle	1				[27]	n.d.
Seychelles	n.d.						
Sierra Leone	n.d.						r.n.
Somalia	n.d.						
South Africa	Rock Hyraxes	2	x	x		[28]	+ (2017)
	Dog	1		x		[29]	
	Sheep	197			x	[30]	
South Sudan	n.d.						
Sudan	Goat	2				[31]	+ (2004)
		3		x		[32]	
		1	x	x		[33]	
	Cattle					[34,35]	
		23			x	[36]	
		13			x		
		11		x			
		25			x	[37]	
		4		x		[38]	
Swaziland	n.d.						
Tanzania	Goat	21			x	[39]	n.d.
	Cattle	11			x		
Togo	n.d.						
Tunisia	n.d.						+ (2002)
Uganda	Cattle	48	x	x		[40]	n.d.
	Cattle	11	x		x	[41]	
	Cattle	35			x	[42]	
Zambia	Sheep	16		x	x	[43]	r.n.
Zimbabwe	n.d.						

n.d.: no data available; r.n.: reported negative after surveillance; S is suspected cases.
